# Is the Interplay between Epigenetic Markers Related to the Acclimation of Cork Oak Plants to High Temperatures?

**DOI:** 10.1371/journal.pone.0053543

**Published:** 2013-01-11

**Authors:** Barbara Correia, Luis Valledor, Mónica Meijón, José Luis Rodriguez, Maria Celeste Dias, Conceição Santos, Maria Jesus Cañal, Roberto Rodriguez, Glória Pinto

**Affiliations:** 1 Department of Biology & Centre for Environmental and Marine Studies, University of Aveiro, Aveiro, Portugal; 2 Epiphysage Research Group, Biology of Organisms and Systems Department, University Institute of Biotechnology & University of Oviedo, Oviedo, Spain; 3 Molecular Systems Biology Department, University of Vienna, Vienna, Austria; 4 Gregor Mendel Institute of Plant Molecular Biology, Austrian Academy of Sciences, Vienna, Austria; National Taiwan University, Taiwan

## Abstract

Trees necessarily experience changes in temperature, requiring efficient short-term strategies that become crucial in environmental change adaptability. DNA methylation and histone posttranslational modifications have been shown to play a key role in both epigenetic control and plant functional status under stress by controlling the functional state of chromatin and gene expression. Cork oak (*Quercus suber* L.) is a key stone of the Mediterranean region, growing at temperatures of 45°C. This species was subjected to a cumulative temperature increase from 25°C to 55°C under laboratory conditions in order to test the hypothesis that epigenetic code is related to heat stress tolerance. Electrolyte leakage increased after 35°C, but all plants survived to 55°C. DNA methylation and acetylated histone H3 (AcH3) levels were monitored by HPCE (high performance capillary electrophoresis), MS-RAPD (methylation-sensitive random-amplified polymorphic DNA) and Protein Gel Blot analysis and the spatial distribution of the modifications was assessed using a confocal microscope. DNA methylation analysed by HPCE revealed an increase at 55°C, while MS-RAPD results pointed to dynamic methylation-demethylation patterns over stress. Protein Gel Blot showed the abundance index of AcH3 decreasing from 25°C to 45°C. The immunohistochemical detection of 5-mC (5-methyl-2′-deoxycytidine) and AcH3 came upon the previous results. These results indicate that epigenetic mechanisms such as DNA methylation and histone H3 acetylation have opposite and particular dynamics that can be crucial for the stepwise establishment of this species into such high stress (55°C), allowing its acclimation and survival. This is the first report that assesses epigenetic regulation in order to investigate heat tolerance in forest trees.

## Introduction

Plants necessarily experience changes in temperature during their life cycle. A diversity of cellular targets is greatly affected by atypically high temperatures that can induce a re-setting of physiological, biochemical and molecular programs and affect plant growth and performance [1]–[3]. Epigenetic modifications in the genome can be induced by environmental signals, and thus, the single genome in a plant cell gives rise to multiple epigenomes in response to different environmental cues [4]–[6]. The control of gene expression based on chromatin organization rather than on primary DNA sequence information is referred to as epigenetics [7]. Epigenetic modifications occur without changing original nucleotide sequence and can be achieved on several interdependent levels that include covalent modifications of DNA and histones [8], [9].

A number of studies have shown that DNA methylation and histone posttranslational modifications play a key role in epigenetic control and plant functional status under stress (e.g. [4], [6], [8], [10], [11]) by controlling the functional state of chromatin and gene expression [12]–[15]. These epigenetic marks are generated fast, can be transmitted across cell divisions (meiotically and mitotically) and can also be reversed, providing a way to confer plasticity in the plant response and temporary “memory” strategies [8], [16]. Experiments inquiring this subject in *Arabidopsis* showed that prolonged heat stress (37°C, 42°C) induces a transient release of gene silencing [17], [18], but once a plant is removed from stress, gene expression is re-established within 48 hours.

Histone acetylation and DNA methylation can activate or repress transcription by generating “open” or “closed” chromatin configuration [19]–[21]. Thus, open chromatin increases the accessibility of the genome to transcription machinery, while closed chromatin represses gene expression by limiting the accessibility [19]. Configuration of chromatin at specific loci also controls somatic homologous recombination; heat stress affects genetic stability through chromatin remodelling, altering accessibility of DNA for repair and recombination [17].

It has long been suspected that a link exists between heat stress, chromatin remodelling, and epigenetic regulation of gene expression but further study is required to confirm the existence and nature of such a link [17]. The work of Kumar and Wigge [22], for example, identified histone H2A as a thermosensor in *Arabidopsis* and revealed a direct link with DNA methylation. Whilst significant progress has been made in understanding the physiological, cellular and molecular mechanisms of plant response to environmental stress factors [1] our understanding of how plants cope with climate challenges is still very limited. Such insight is required to understand heat-induced epigenetic processes. In fact, because these epigenetic traits exhibit characteristic dynamics during growth and development they may be of crucial importance in exploring and understanding adaptation related processes throughout the life cycle of trees, particularly in response to stress. There is an urgent need to determine the adaptive potential of forest trees given their importance in ecosystem functioning and the associated ecological and economic services they provide.

This topic was addressed in *Quercus suber* L. (cork oak) plants that were demonstrated to be extremely tolerant to elevated temperatures [23], [24]. In the field, cork oak plants can be exposed to temperature near 40–45°C (in shade) and experience daily stress [23], [25]. Cork oak is widely distributed and withstands a variety of climates with contrasting temperatures and rainfall [26], making summer drought and high temperatures clear selective agents [27]. These factors are thought to be among the most significant in the increasing mortality of forests in response to global climate changes [28].

Cork oak's ability to acclimate to stress conditions may be an important factor in the tolerance of this species to high summer temperatures [23], [24]. In the Mediterranean area, cork oak is of great ecological and economic importance. It is expected to be severely affected by climate change due to the increased intensity and duration of the drought and heat periods expected for this region [27].

Few studies have been conducted which test the effect of high temperatures in *Quercus* and the majority of those which have been conducted focused on the photosynthetic apparatus and volatile organic compounds production [23], [25], [29]–[31]. At the molecular level information is still scarcer [32] particularly for such high temperatures. Epigenetic changes that may occur under such conditions therefore remain largely unexplored.

To test the hypothesis that the epigenetic code could be related to heat stress tolerance in cork oak, DNA methylation and acetylated histone H3 (AcH3) levels were monitored during cumulative high temperature stress from 25°C to 55°C (in 10°C steps). In addition, the spatial distribution of these modifications was followed by immunolocalization in order to validate the results and analyse the possible correlation between heat stress tolerance and the studied epigenetic marks. The aim of this work was to collect, for the first time, epigenetic knowledge related to heat tolerance in cork oak and contribute to the current understanding of epigenetic control of heat acclimation in forest trees.

## Materials and Methods

### Plant material and experimental design

8-month-old cork oak plants were acquired from forest plant producer ANADIPLANTA (located in Central Portugal) and transferred from semi controlled greenhouse conditions to a climate chamber for a 2 weeks acclimation period. The climate chamber environment was kept constant (air temperature = 25°C; relative humidity = 60–70%; photosynthetic photon flux density = 250 µmol m^−2^ s^−1^; watering = field capacity; photoperiod = 16 h).

During the experimental treatment, relative humidity, irradiance, watering and photoperiod were held constant, while air temperature was gradually increased by 10°C every 3 days from 25°C to 55°C, peak temperature was maintained for 3 hours. Minimum daily temperature was 20°C during the 8 night hours.

Sampling occurred on the third day during peak heat hours (around 12 a.m.) for each temperature (25°C, 35°C, 45°C and 55°C). Fully expanded leaves were collected from each treatment, frozen in groups of 5 individuals (pools) in liquid nitrogen and stored at −80°C for subsequent analyses. Leaf sections were also fixed in paraformaldehyde for further immunohistochemical detection. Fresh leaf samples were used for determination of relative electrolyte leakage.

### Percentage of survival, visual leaf damage and determination of relative electrolyte leakage

The percentage of survival plants and visual leaf damage were recorded for each temperature. To get more information on the cell-membrane damage caused by heat stress, the membrane permeability of the leaves was measured by electrolyte leakage. Leaves were rinsed three times with deionized water to remove surface-adhered electrolytes, then placed in tubes containing 20 mL of deionized water and incubated at 25°C on a shaker. Twenty four hours later, the electrical conductivity of the bathing solution (C1) was determined using a conductivity instrument (pH 340/ION, WTW, Germany). The tubes were then autoclaved at 100°C for 25 min and subsequently maintained at 25°C. Finally, total electrical conductivity (C2) was measured and electrolyte leakage was calculated using the following equation: relative electrolyte leakage (%) = (C1/C2)×100. Eight biological samples were analysed.

### Nuclei isolation

Nuclei were isolated from 500 mg of frozen leaves using the protocol described by Haring et al. [33] with the following modifications: samples were transferred to 12 mL tubes containing 8 mL ice-cold cell isolation buffer (10 mM Tris pH 8.0, 400 mM sucrose, 10 mM Na-butyrate, 0.1 mM phenylmethylsulfonyl fluoride (PMSF), 5 mM β-mercaptoethanol, protease inhibitors 1 µg/mL) and filtered through 3 layers of Miracloth into a new ice-cold 12 mL tube. After centrifuging the filtrate (3000×g, 15 min, 4°C), the supernatant was removed and the pellet was resuspended in 5 mL ice-cold nuclei isolation buffer A (10 mM Tris pH 8.0, 250 mM sucrose, 10 mM Na-butyrate, 10 mM MgCl_2_, 1% v/v Triton X-100, 0.1 mM PMSF, 5 mM β-mercaptoethanol, protease inhibitors 1 µg/mL) and incubated for 10 minutes on ice. The solution was centrifuged (3000×g, 15 min, 4°C), the resulting supernatant was removed and the pellet was resuspended and incubated in 5 mL ice-cold nuclei isolation buffer until pellet was light green. After centrifugation (3000×g, 15 min, 4°C) the supernatant was removed and the pellet was resuspended in 8 mL ice-cold nuclei isolation buffer B (10 mM Tris pH 8.0, 1.7 M sucrose, 10 mM Na-butyrate, 2 mM MgCl_2_, 0.15% v/v Triton X-100, 0.1 mM PMSF, 5 mM β-mercaptoethanol, proteinase inhibitors 1 µg/mL). The solution was centrifuged (3000×g, 15 min, 4°C), the supernatant was removed and the isolated nuclei were kept at −80°C until DNA or protein extraction.

### Global nuclear DNA methylation

Nuclear DNA was extracted from the pre-isolated nuclei following the procedure described by Thomas et al. [34], modified in the ensuing points: nuclear pellets were transferred to a 2 mL tube with 1.25 mL buffer 1 (0.25 M NaCl, 0.2 M Tris-HCl pH 7.6, 0.05 M Na_2_EDTA pH 8.0, 2.5% v/v β-mercaptoethanol, 2.5% w/v polyvinylpyrrolidone (MW 40.000)). The mixture was briefly mixed by vortexing and centrifuged (2600×g for 5 min at 4°C). After removing the supernatant, pellet was resuspended in 500 µL buffer 2 (0.05 M NaCl, 0.2 M Tris-HCl pH 8.0, 0.05 M Na_2_EDTA pH 8.0, 2.5% v/v β-mercaptoethanol, 2.5% w/v polyvinylpyrrolidone (MW 40.000), 3% sarkosyl) and incubated at 37°C for 30 min with gently shaking. After adding an equal volume of chloroform/isoamyl alcohol, centrifuging and collecting the aqueous phase in a new tube, 350 µL of cold isopropanol was added and slowly mixed to precipitate DNA. DNA was collected and transferred to 500 µL 70% ethanol. Ethanol-DNA mixture was centrifuged (19000×g for 10 min at 4°C), supernatant was discarded and after completely dry, DNA pellet was resuspended in 100 µL _dd_H_2_O. DNA suspensions were purified using phenol-chloroform solution and completely air-dried DNA pellets were resuspended in 12 µL _dd_H_2_O. An aliquot of each extracted sample was used to evaluate DNA concentration and integrity and to detect residual RNA.

DNA hydrolysis and global DNA methylation analysis were performed by high performance capillary electrophoresis (HPCE) according to previously described by Hasbún et al. [35]. Four pools of five samples and three analytical measurements were analysed at each experimental situation. The methylation content of each DNA sample was quantified as: 5-mdC peak area×100/(dC (deoxycytidine) peak area + 5-mdC peak area).

### Methylation-sensitive random-amplified polymorphic DNA (MS-RAPD)

Genomic DNA was extracted from 75 mg of frozen leaves with a plant genomic DNA extraction kit (DNeasy Plant Mini Kit, Qiagen, Germany) according to the manufacturer's instructions. DNA yield and purity were assessed by spectrophotometry (as described in Valledor et al. [36]) and gel electrophoresis on agarose gel by direct comparison with phage λ DNA.

To study the epigenetic changes in specific DNA sequences it was used MS-RAPD, a modification of the original RAPD technique which is closer to MSAP (methylation sensitive amplified polymorphisms) analysis. All steps in the protocol were carefully standardized and thoroughly described in order to ensure technical reproducibility.

In a first step, two methylation-sensitive isoschizomers (*Hpa*II and *Msp*I) were used in parallel to digest the DNA.

After digestion, a standard RAPD procedure was used to amplify the restriction fragments. In the first reaction, 250 ηg of each extracted DNA was added to a restriction mixture containing: 1× restriction buffer and 10/20 U restriction endonuclease to a final volume of 30 µL, one mixture per endonuclease (*Hpa*II/*Msp*I; all endonucleases and buffers were supplied by New England Biolabs, USA). Restriction mixtures were then incubated at 37°C overnight. The reaction was stopped by placing the tubes on ice and restriction was checked by agarose electrophoresis. PCR amplification was performed according to Cocconcelli et al. [37]: 20 µL reaction mixtures containing: 12–14 ng DNA, 1× PCR buffer (Invitrogen, USA), 3.5 mM MgCl_2_, 75 pM dNTP, 0.25 mM each primer, 1 U Taq polymerase (Invitrogen, USA). In a preliminary work, a total number of 20 primers was tested (one ‘arbitrary’ primer set – Operon OPH). From this preliminary test, only the 10 most polymorphic primers were selected and used in the current manuscript (see table 1).

**Table 1:** Sequences of primers used in MS-RAPD protocol.

Name	Sequence
OPH-03	AGACGTCCAC
OPH-04	GGAAGTCGCC
OPH-05	AGTCGTCCCC
OPH-07	CTGCATCGTG
OPH-08	GAAACACCCC
OPH-10	CCTACGTCAG
OPH-13	GACGCCACAC
OPH-18	GAATCGGCCA
OPH-19	CTGACCAGCC
OPH-20	GGGAGACATC

Amplification was performed in a thermo cycler in 96-well plates. The cycle conditions were as follows: initial denaturation at 94°C for 5 min, followed by 45 cycles 94°C for 1 min, annealing temperature of 29°C for 1 min and extension at 72°C for 2 min. A ramp of 1.5 min was used between annealing (29°C) and elongation (72°C). None of the primers used in this study revealed any change in DNA fingerprint within the samples when RAPD analysis was performed to undigested genomic DNA, so all of the quantified changes are the results of changes in DNA methylation and not in sequence-differences between individuals.

The products of RAPD assay were resolved on 1.75% agarose gels. Interpretation of MS-RAPD bands followed the representation of MSAP (methylation sensitive amplified polymorphisms) detected by *Hpa*II/*Msp*I endonuclease digestion according to Valledor et al. [38] and appearance-disappearance of bands was used to study the variation of methylation which could be classified into two categories: *de novo* methylation and demethylation events. Four pools of five samples were analysed. The complete protocol was performed two unattached times to ensure the reliability of the results. Only consistent bands between batches were considered for analysis.

### Protein extraction

Proteins were extracted from the pre-isolated nuclei, following part of the procedure described by Shechter et al. [39]. In brief, nuclear pellets were transferred to a fresh 1.5 mL tube with 400 µl 0.8N H_2_SO_4_. The suspensions were vortexed until clumps were dissolved and incubated on rotator, for 30 min. After this time, the solutions were sonicated and then centrifuged (15000×g for 10 min at 4°C) to remove nuclear debris. Supernatants were transferred to a fresh 1.5 mL tube and they were added 140 µL 100% trichloroacetic acid (TCA). Suspensions were incubated on ice for 30 min and then centrifuged at 15000×g during 10 min at 4°C. Supernatants were discarded and then pellets were washed with 1 mL cold-acetone. Pellets were recovered by centrifugation (15000×g, 10 min, 4°C) and supernatants were discarded. This washing step was repeated twice. Then the protein pellets were air-dried at room temperature and later dissolved in appropriate volume of protein rehydration buffer (8 M urea, 2% w/v 3[(3-cholamidopropyl) dimethylammonio]-propanesulfonic acid (CHAPS), 8 mM dithiothreitol (DTT), 0.5% v/v ampholytes).

### Protein blot

Protein gel blot analysis was performed as described by Valledor et al. [38]. In brief, proteins and standards were separated by electrophoresis in 13.5% w/v acrylamide SDS gels (Mini-PROTEAN II Multi-Casting Chamber, BIO-RAD, USA) and then transferred by electroblotting (350 mA for 2 hours) to Immobilon membranes (Millipore Corp., USA). For the immunodetection, the membranes were blocked overnight at 4°C in 2% w/v powdered skimmed milk in phosphate-buffered saline (PBS) containing 0.5% v/v Tween 20. Then, the membranes were incubated with primary and secondary polyclonal antibodies diluted 1∶2000 and 1∶1000 in blocking solution for 2 and 1 hours, respectively. The used primary antibodies were anti-acetyl-Histone H3 (anti-AcH_3_) (rabbit, Millipore Corp., USA) and anti-actin (rabbit, Chemicon, USA). Latter secondary antibodies (anti-rabbit), coupled to alkaline phosphatase, were used in a dilution of 1∶5000 and signals were revealed in a nitroblue tetrazolium and bromo-chloro-indolyl-phosphate (NBT-BCIP) mixture. Anti-actin was used as a control for loading normalization.

Densimetric measurements were taken after immunodetection using Kodak.1D v 3.6 Scientific Imaging Systems (USA). Abundance index was calculated as follows: protein band intensity/actin band intensity. Four pools of five samples were analysed.

### Immunohistochemical detection

The immunolocalization was carried out according to the procedure described by Valledor et al. [38]. Half cross sections of leaf were fixed in 4% paraformaldehyde, 1% β-mercaptoethanol in PBS overnight at 4°C and sectioned at a 50 µm thickness using a cryomicrotome Leica CH 1510-1 (Leica Microsystems, Germany). The samples were mounted on slides coated with APTES (3-aminopropyltriethoxysilane; Sigma, USA). The leaf sections were dehydrated and then rehydrated through ascending and descending series of ethanol, respectively. The leaf sections were incubated in 2% cellulase in PBS for 45 min at room temperature and denatured in 2N HCl for 30 min. The permeabilised sections were blocked in 5% bovine serum albumin (BSA) in PBS for 10 min and incubated 1 h with mouse antibody anti-5-methylcytidine (anti-5-mdC, Eurogentec, Belgium) diluted 1∶50 in 1% blocking solution or with rabbit antibody anti-acetylated-Histone H3 (anti-AcH_3_, Millipore Corp., USA) diluted 1∶25. Non-bound antibodies were washed with 0.1% Tween 20 in PBS. Alexa Fluor 488-labelled anti-mouse polyclonal antibody (Invitrogen, USA) diluted 1∶25 was used as secondary antibody for the 5-mdC detection and Alexa Fluor 488-labelled anti-rabbit polyclonal antibody (Invitrogen, USA) for the acetylated H3 histone detection. The slides were counterstained with DAPI (4′, 6-diamidino-2-phenylindole; Fluka, USA). Fluorescence was visualized using a confocal microscope (Leica TCS-SP2-AOBS; Leica Microsystems, Germany). Three biological samples were analysed for each treatment and multiple 3D image stacks were acquired of each leaf. 3D images of whole leaf were reconstructed and maximal projection was performed using Leica Software (LCS c2.5. Leica Microsystems, Germany).

### Statistical analysis

Statistical analyses were conducted with SigmaPlot statistical software package v. 11 (Systat, Germany) for Windows. Data from electrolyte leakage, global DNA methylation and relative abundance index of AcH3 were subjected to an analysis of variance using One Way Anova. When data were statistically different Anova test was followed by a Holm Sidak test (p<0.05).

## Results

**Figure 1:**
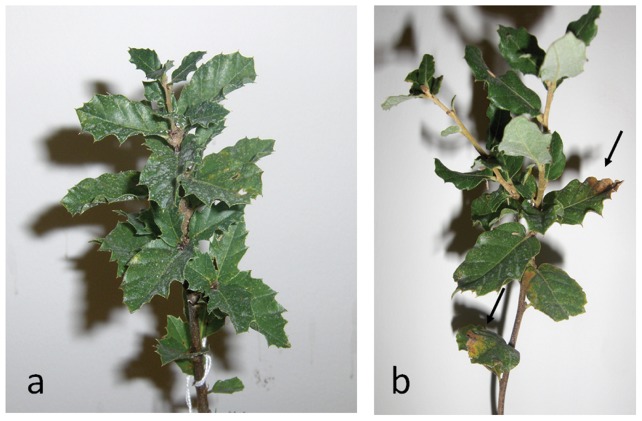
*Quercus suber* plants used during the experience. a) Plants at 25°C; b) Plants after three days at 55°C with clear signals of stress (brown areas, black arrows).

Percentage of survival, visual leaf damage and electrolyte leakage were measured in plants exposed to temperature increases from 25 to 55°C (in 10°C steps). All plants survived to 55°C. Nevertheless, visual leaf damage was evident at 55°C, where it was possible to detect brown spots (Fig. 1). No morphological signal of stress was detected below this temperature.

**Figure 2:**
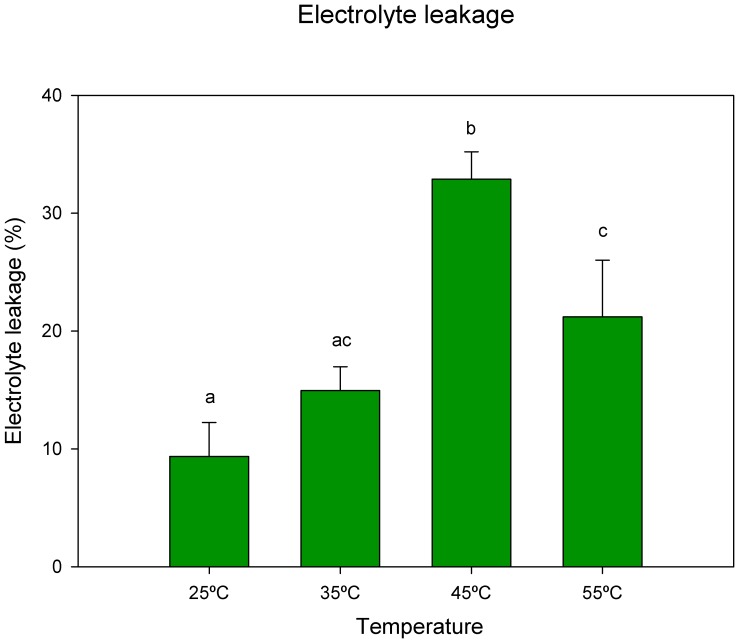
Electrolyte leakage in leaves of cork oak plants exposed to heat stress. Data are means ± SD. Bars with different letters indicate significant differences between treatments (p<0.05).

Cumulative heat stress led to higher electrolyte leakage (an indirect parameter of membrane rupture) that increased significantly between 35°C–45°C. The maximum degradation was observed at 45°C. At 55°C electrolyte leakage percentage decreased significantly (Fig. 2).

**Figure 3:**
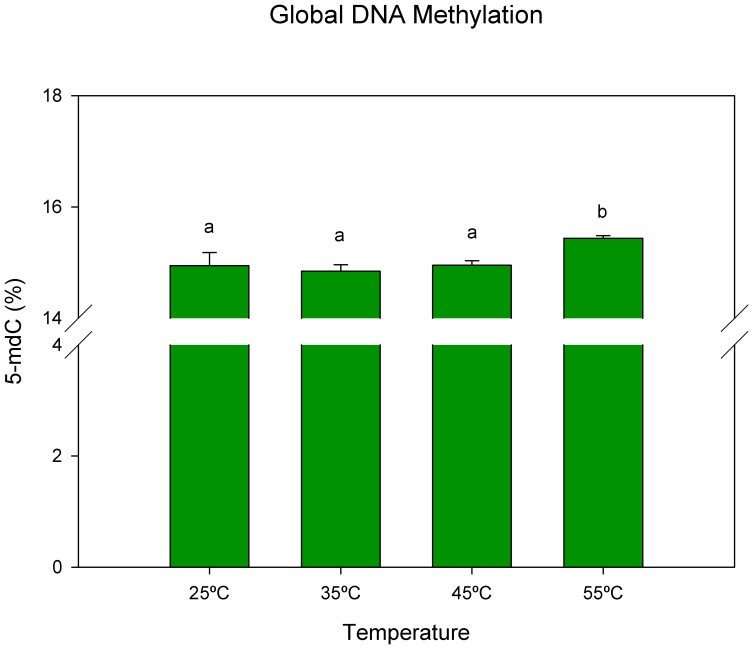
Global genomic DNA methylation percentages in leaves of cork oak plants exposed to heat stress. Data are means ± SD. Bars with different letters indicate significant differences between treatments (p<0.05).

DNA methylation analysed by HPCE (Fig. 3) revealed no significant alterations in global DNA methylation with increasing heat stress until 45°C, but a significant increase at 55°C (p<0.05).

**Figure 4:**
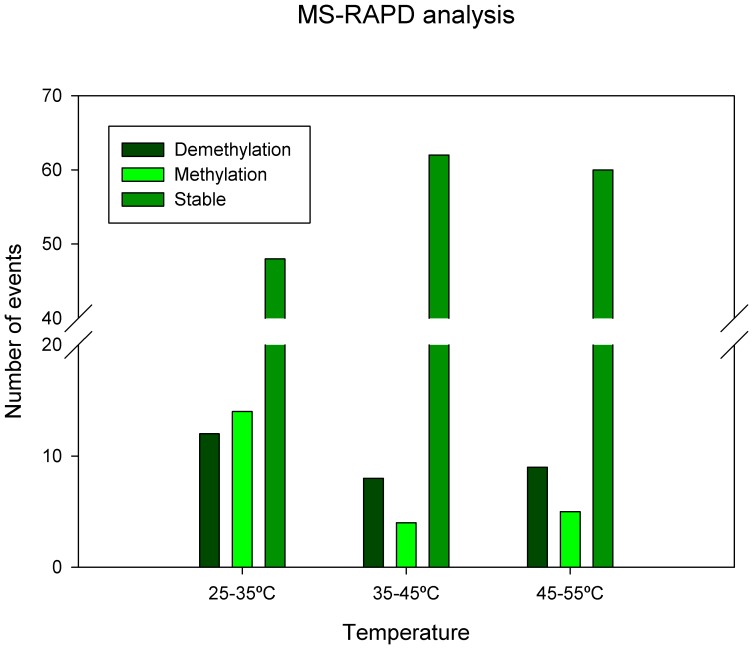
MS-RAPD profiles of cork oak leaves exposed to heat stress. Interpretation of MS-RAPD bands followed the representation of MSAP (methylation sensitive amplified polymorphisms) detected by *HpaII*/*MspI* endonuclease digestion according to Valledor et al. (2010). Appearance-disappearance of bands was used to study the variation of methylation between treatment steps (25°C to 35°C, 35°C to 45°C and 45°C to 55°C). Fragment analysis allowed the classification into two categories: *de novo* methylation and demethylation events.

The MS-RAPD analysis showed different methylation patterns at specific sequences. Seventy four bands were analysed: from 25°C to 35°C (12 demethylation and 14 *de novo* methylation events); from 35°C to 45°C (8 demethylation and 4 *de novo* methylation events); finally 9 demethylation and 5 *de novo* methylation events were measured from 45°C to 55°C (Fig. 4). These results showed a high dynamic number of events between 25°C and 35°C in comparison the others increase steps of temperature.

**Figure 5:**
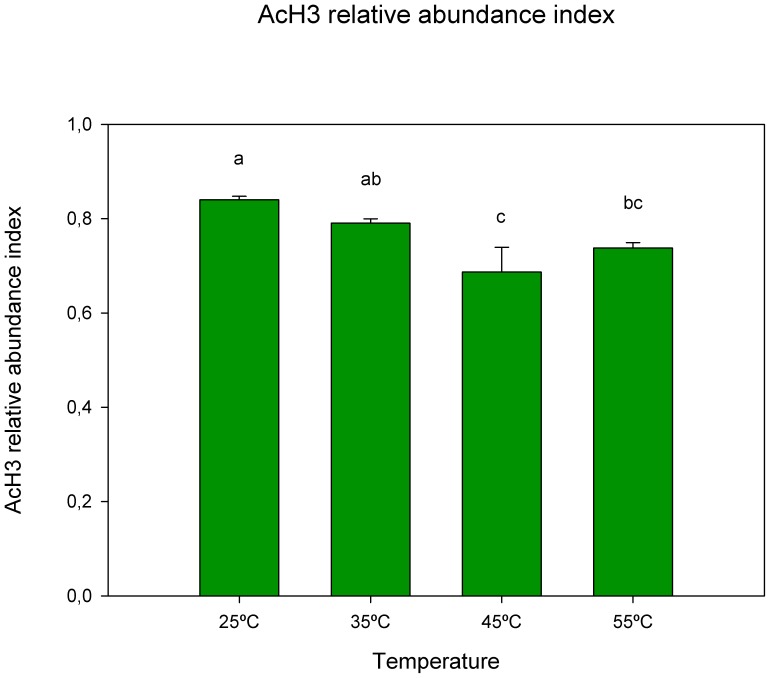
Western blot analysis showing the quantification of AcH3 in leaves of cork oak plants exposed to temperature increase. Values represent relative abundance index calculated as: AcH3 band intensity/actin band intensity. Data are means ± SD. Bars with different letters indicate significant differences between treatments (p<0.05).

Protein extracts from cork oak leaves exposed to 25°C, 35°C, 45°C and 55°C revealed single bands of approximately 17 and 43 kDa for acetylated histone H3 (AcH3) and actin, respectively. The abundance index of AcH3 decreased from 25°C (0.840) until 45°C (0.790 at 35°C and 0.686 at 45°C), and presented a slightly superior value at 55°C (0.737) (Fig. 5).

**Figure 6:**
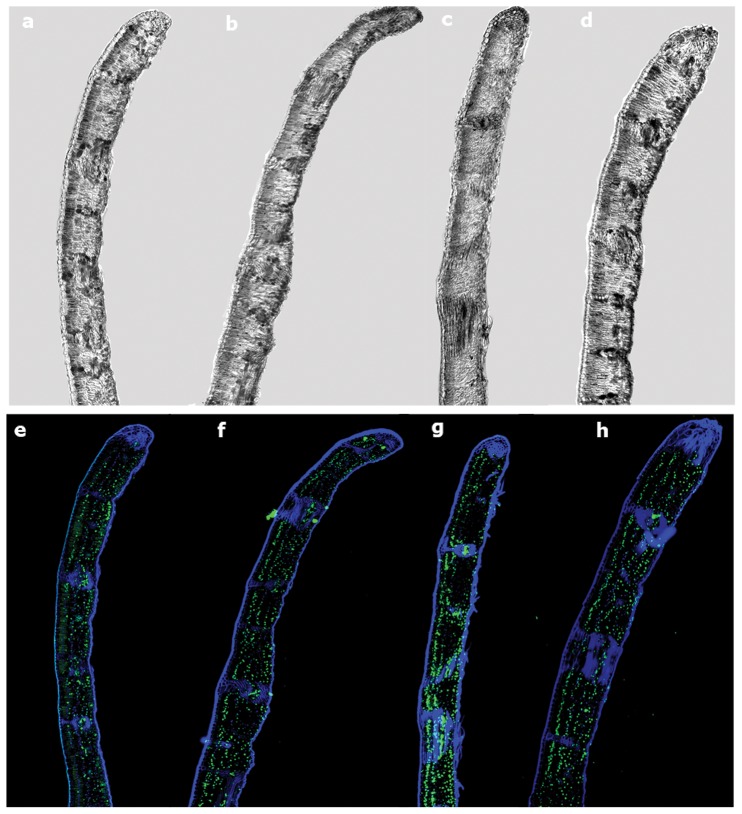
Immunodetection of 5-mdC in sections of cork oak leaf in transversal axis using a confocal microscope. Differential Interference Contrast (DIC) of leaf section at (a) 25°C; (b) 35°C; (c) 45°C; (d) 55°C. 5-mdC labeling: DAPI (blue signals) superposition and 5-mdC (green signals) of leaf section at (e) 25°C; (f) 35°C; (g) 45°C; (h) 55°C.

**Figure 7:**
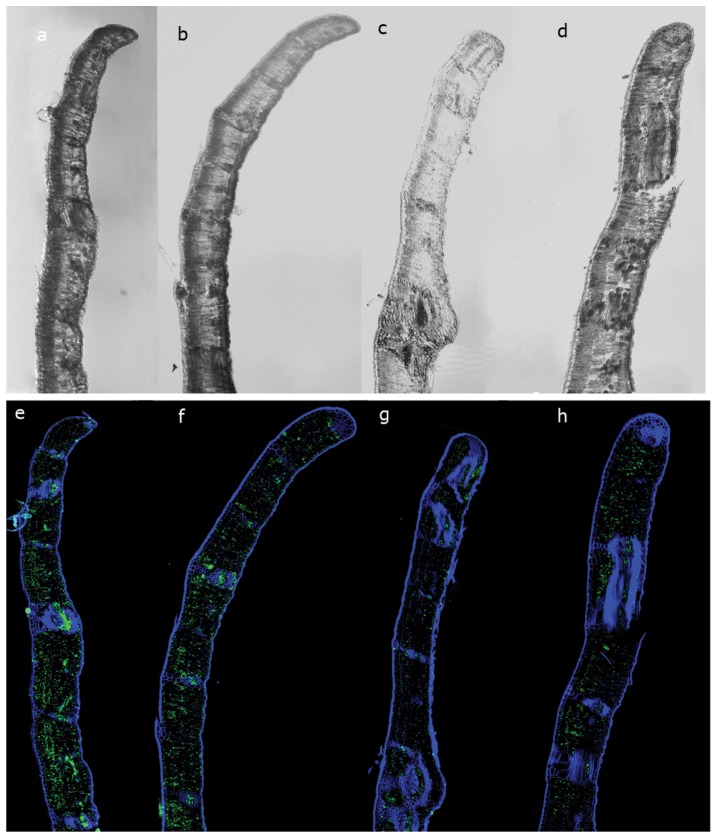
Immunodetection of AcH3 in sections of cork oak in transversal axis using a confocal microscope. Differential Interference Contrast (DIC) of leaf section at (a) 25°C; (b) 35°C; (c) 45°C; (d) 55°C. AcH3 labeling: DAPI (blue signals) superposition and AcH3 (green signals) of leaf section at (e) 25°C; (f) 35°C; (g) 45°C; (h) 55°C.

The immunolocalization analysis showed different 5-mdC levels and spatial distribution in leaves in response to heat stress increase (Fig. 6). At 25°C 5-mC was equitably distributed over the tissues of the sample (Fig. 6e). An increasing in intensity of methylated cytosine at 35°C was observed, occupying the four cellular layers (Fig. 6f). At 45°C there is a clear redistribution of the methylated cytosine; such redistribution is much less evident in the spongy parenchyma and more concentrated near palisade parenchyma and vascular vessels (Fig. 6g). At 55°C the distribution occurred in similar pattern as to that at 35°C but with much more methylated nuclei (Fig. 6h). The histone AcH3 signal presented an opposite pattern in the immunolocalization analysis (Fig. 7): the intensity declined from 25°C to 45°C, showing a slight increase at 55°C (Fig. 7h).

## Discussion

Forest trees are long-lived organisms and, as masters of adaptation, they can tolerate a very wide range of growing conditions and extreme seasonal changes. Despite the short-term heat stress experiment that was implemented, the survival of all 8 month-old cork oak plants to 55°C was remarkable and strengthens the reports of high thermotolerance within this species by other authors [23].

This tolerance highlights the well-designed adaptive mechanisms present in cork oak plants, but despite of this tolerance, it is underlined that a period of stress is necessary to trigger the physiological responses. For example, when focusing on membrane stability it was visible an increase of electrolyte leakage until 45°C. After that, a decrease getting closer to the values found for 35°C was observed. This unexpected profile suggests acclimation even at this high temperature. This could be explained by a physiological and biochemical re-setting in response to stress, which allowed stabilization and protected the membranes. The increasing expression of heat shock proteins and proline at 55°C are probably part of the explanation (unpublished data).

It is clear that gene control plays important roles defining the tissue adaptation to stress. To achieve the new morphological and physiological status, genes must be regulated to express only in certain cells and situations. Changes in epigenetic marks accompany morphological and physiological changes in trees in a wide range of processes such as phase change, aging, flowering time, organ maturation [35], [38], [40], [41] and also variation in natural plant populations [9].

Gene expression driven by developmental and stress cues often depends on DNA methylation and histone posttranslational modifications. These epigenetic mechanisms are crucial to adapt plant responses to stress that result in short-term acclimation [11]. The performed analysis revealed that heat stress induced covalent modifications such as DNA methylation and histone acetylation.

It has previously been shown that cytosine methylation is altered in response to environmental stimuli throughout the genome at specific loci [42]. MS-RAPD results pointed dynamic methylation-demethylation patterns over stress. While HPCE quantifies all cytosines present in the genome, MS-RAPD can only discriminate methylation of cytosines present in specific CCGG sequence. According to the literature, these CCGG sites are present in a small part of the genome of the plants, and are frequently located in gene promoter regions, where cytosine methylation usually implies repressive chromatin in gene promoters and repression of gene transcription [6]. MS-RAPD analysis showed that the first temperature ramp (25°C–35°C) was highly dynamic with highest number of methylation and demethylation events, and higher rate of *de novo* methylation than demethylation. This fact suggests that, in an initial stressing condition, cork oak cell reorganizes its DNA structure and compaction grade by methylation-demethylation mechanisms to promptly regulate gene expression, activating effective defence mechanisms to overcome the stressful conditions. After that (35°C–45°C; 45°C–55°C) a new baseline is achieved at DNA methylation profile that is indicated by an increased number of constant bands between temperatures. When looking at genome level by quantification by HPCE, it was observed that DNA methylation was stable until 45°C, showing a marked increment after that. Supporting these results, in cold treated maize leaves DNA methylation level increased [43], and in long time pine trees exposed to radiation the level of DNA methylation also increased, explained as being a possible mechanism of adaptation to radiation [44]. Tan [42] and Pecinka et al. [17] both stressed the importance of increasing methylation of transposable elements that is frequently associated with transcriptional gene silencing.

The immunohistochemical detection of 5-mdC came upon these results, highlighting an increase in DNA methylation over stress. A re-distribution was noticed at 45°C, with an accumulation of 5-mdC near both epidermis and vascular vessels. This re-distribution concurred with membrane stability results that showed the highest values of electrolyte leakage at 45°C. The decrease at 55°C might be related with the acclimation process. This may be explained by the activation of other molecular pathways related to membrane stability.

Despite the global increasing methylation given by HPCE and immunolocalization of 5-mdC, the MS-RAPD results demonstrated that specific demethylation simultaneously occurred in many loci. These findings are supported by other works, such as Boyko et al. [45] who found that although a hypermethylation in genes and promoters in stressed *Arabidopsis* progeny is evident, many loci in the genome are hypomethylated.

The acetylation states of histone H3 decreased under stress as evidenced in Protein blot and by immunolocalization. Tsuji et al. [46] found that submergence induced histone H3 acetylation in *Oryza sativa* and concluded that this histone modification was correlated with enhanced expression of ADH1 and PDC1 genes under stress. These differences lead us to speculate that deacetylated H3 in *Q. suber* is responsible for repressive chromatin in gene promoters and repression of gene transcription. Modifications in histone H3 were also observed in heat acclimation in animals showing a conserved mechanism both in plants and in animals [47].

The opposite labelling profile of 5-mdC and AcH3 observed here during the increasing heat stress indicates cooperation of both epigenetic mechanisms, demonstrating the well coordinated and interdependent relations between the explored epigenetic markers as reported by other authors [20], [48]. The layout of these marks in immunolocalization shows a differential distribution in the cross section evaluated. Accordingly, this technique has successfully been used to inquiry the differentiation of specific tissues like in flowering development [49], [50] or maturation [38].

The results here reported proved that cork oak leaves experience interrelated and specific DNA methylation and histone H3 acetylation changes due to the elevated temperature conditions. Such interplay can be crucial for the stepwise establishment of this species into such high stress (55°C) that allow the acclimation and survival. Up to authors knowledge the only report that analysed the distribution of epigenetic marks in cork oak was assessed in the nuclei of mature pollen cells [51]. Here the high level of DNA methylation associated with histone modifications in the vegetative nucleus indicated a high potential for transcriptional silencing according to the apparent chromatin silencing of the generative nucleus [51].

The evolutionary impact of such regulation could rely on a “memory” of stressful conditions faced by ancestor leading to a better adaptation of the progeny. The existence of this memory has still to be fully established for perennial species. Recently, a temperature-dependent epigenetic memory was reported for Norway spruce where the temperature of embryo development had later influence on bud timing phenology and gene expression [16], [52]. Insights into epigenetics variation will contribute to the understanding of adaptive plant response in a climate change scenario. The question of whether the perceived environmental cues were memorized by cork oak plants (in a form that is maintained even when the stimulus is removed) now provides an interesting avenue for future research.

Further studies are needed, to specifically link physiology and molecular aspects and aid understanding heat tolerance in forest key trees under conditions such as those present in the Mediterranean region. The results published here open new perspectives in a non model woody species and help us to understand, for the first time, epigenetic regulation in response to high temperatures.
